# The controlling nutritional status score and clinical outcomes in patients with heart failure: Pool analysis of observational studies

**DOI:** 10.3389/fcvm.2022.961141

**Published:** 2022-07-25

**Authors:** Xian-Wen Huang, Jian-Jing Luo, Beatrice Baldinger

**Affiliations:** ^1^Department of Emergency Intensive Care Medicine, The People's Hospital of Bao'an, Shenzhen, China; ^2^Department of internal medicine, Zhaoqing Medical College, Zhaoqing, China; ^3^Department of cardiology, Bern University Hospital, University of Bern, Bern, Switzerland

**Keywords:** heart failure, malnutrition, prognosis, risk, the controlling nutritional status

## Abstract

**Background and aims:**

Malnutrition is very common in patients with heart failure (HF) and is associated with a worse clinical outcome. The Controlling Nutritional Status (CONUT) score is an easily derived index for the evaluation of malnutrition. This study aimed to evaluate the association between the CONUT score and the prognosis in patients with HF.

**Methods and results:**

Electronic databases were searched for potential studies from inception up to February 15, 2022. Observational cohort studies included adult participants with HF, and reported the associations between the CONUT score and the adjusted relative risk (RR) of all-cause mortality, and patients with composite major adverse cardiac outcomes (MACEs) were included. We finally included 18 studies comprising 12,532 participants with HF for analysis. The median age of the patients was 70.5 years old, and 35.4% were women. After a median follow-up duration of 32.5 months, patients with HF with a higher CONUT score were associated with a higher risk of all-cause mortality (per 1 increment of the CONUT score: RR, 1.21, 95% CI, 1.13–1.29, *I*^2^ = 68%, *P* for heterogeneity = 0.002) and MACEs (per 1 increment of the CONUT score: RR, 1.14, 95% CI, 1.06–1.23, *I*^2^ = 81%, *P* for heterogeneity <0.0001) after adjusting for other prognostic factors. When the CONUT score was divided into the normal nutritional status and malnourished status, malnourished patients with HF were associated with increased risks of all-cause death (RR, 1.61, 95% CI, 1.40–1.85, *I*^2^ = 17%, *P* for heterogeneity = 0.29) and MACEs (RR, 2.12, 95% CI, 1.49–3.02, *I*^2^ = 87%, *P* for heterogeneity <0.0001), compared with those with normal nutritional status.

**Conclusions:**

The CONUT score is associated with the clinical outcomes in patients with HF, and can be used as a screening tool of nutritional status in HF to improve prognosis.

## Introduction

Heart failure (HF) is a complex clinical syndrome that results from any structural or functional impairment of the heart. Accompanied by the aging of society and a decrease in mortality of multiple cardiovascular diseases, the prevalence of HF has increased rapidly, which contributed to a growing health burden worldwide (1, 2). Although guideline-directed medical therapy (GDMT) had made great progress in the management of HF, it was still associated with high morbidity and mortality. It had been reported that, in patients hospitalized due to the exacerbation of HF, the composite outcomes (including 1-year mortality and re-hospitalization) were >20% (3, 4). Therefore, new risk stratification markers and treatment methods are still needed to improve the prognosis of HF.

Malnutrition is very common in patients with HF and is associated with a higher risk of mortality and re-hospitalization (5, 6). Early detection of malnutrition in HF would be useful for identifying patients at high risk of poor clinical outcomes and recommending nutritional interventions to improve prognosis (7). Many tools and indexes had been proposed for screening malnutrition; however, no consensus had been made on which to use in patients with HF (5, 8–10).

The Controlling Nutritional Status (CONUT) score, developed by Ignacio et al., (11) had been reported to be one of the most robust markers of nutritional status. It is calculated from a patient's serum albumin, total cholesterol level, and total peripheral lymphocyte count. Therefore, The CONUT score is an immune-nutritional index, which can evaluate the protein reserve, lipid metabolism, and immunocompetence. Recently, studies have shown that malnourished status determined by the CONUT score is associated with worse outcomes in patients with HF (12–16). However, these studies were with small sample size and different patient characteristics, which resulted in inconsistent results in the association between the CONUT score and the clinical outcomes in patients with HF. Based on the inconsistency of previous studies, we conducted a meta-analysis of observational cohort studies to evaluate the association between the CONUT score and the prognosis in HF.

## Methods

We performed the systematic review and meta-analysis according to the recommendations of the MOOSE (Meta-analysis of Observational Studies in Epidemiology) Group (17). Electronic databases, including PubMed, Embase, Google Scholar, the Cochrane Library, and Wanfang, were searched for related studies from inception until February 15, 2022. We developed the search strategies using the terms “Controlling Nutritional Status,” “CONUT,” or “malnutrition” and “heart failure,” “cardiac dysfunction,” or “myocardial dysfunction” and “prognosis,” or “death” or “MACE.” We limited our search to human studies and writing in Chinese or English, and further read the reference lists of the included studies or other systematic reviews to identify potential missing related articles.

Two researchers (X-WH. and J-JL) independently searched the databases and screened the retrieved items. Potentially related studies were reviewed in full text, and the studies' information was extracted into a pre-defined form. We included studies for meta-analysis if there were: (1) observational cohort studies included adult participants (age ≥18 years old); (2) all the participants were diagnosed with HF; (3) the CONUT score was evaluated at baseline status, which was based on serum albumin, lymphocyte count, and total cholesterol measures (range from 0 to 12); (4) the association between the CONUT score (as a continuous or category metric) and the prognostic outcomes of HF were reported in an adjusted model, which was controlling the other related prognostic factors. We excluded those studies if they were: (1) cross-sectional studies; (2) the follow-up evaluation was <3 months; (3) the relative risk (RR) was not adjusted for other confounders, and (4) duplicated publications from identical cohort studies with the same outcomes.

The CONUT score was calculated based on the patients' serum albumin, total cholesterol, and total peripheral lymphocyte levels (Table 1). The range of the CONUT scores is 0 to 12, and a higher score indicated that the patient was with worse nutritional status (11–16). The quality of the included studies was accessed by the NOS (the Newcastle–Ottawa Quality Assessment Scale for cohort studies), which evaluates the selection (four items with one point in each item), comparability (one item with up to two points), and exposure/outcome (three items with one point in each item), respectively (18, 19). Therefore, up to a highest of 9 points can be awarded in NOS. According to previous reports, the included studies were graded as low quality (<4 points), moderate quality (4–6 points) or high quality (≥7 points), respectively (20, 21).

**Table 1:** Parameters for assessment of the CONUT Score.

**Parameter**	**Score**			
Serum albumin (g/ml)	≥3.5	3.0–3.49	2.50–2.99	<2.50
Albumin score	0	2	4	6
Total cholesterol (mg/dl)	≥180	140–179	100–139	<100
Cholesterol score	0	1	2	3
Lymphocytes (count/ml)	≥1,600	1,200–1,599	800–1,199	<800
Lymphocytes score	10	1	2	3

In this meta-analysis, the primary outcome interested was all-cause mortality in patients with HF. The secondary outcome was composite major adverse cardiac outcomes (MACEs), including all-cause mortality and HF hospitalization. We pooled the association between the exposure (CONUT score) and outcomes in multivariable-adjusted statistical models. If multiple statistical models were reported, we used the data that adjusted the most comprehensive confounders for analysis. As the associations between the CONUT score and the interested outcomes were reported in different ways in the included studies (e.g., per 1 increment as a continuous metric; or as normal nutritional/malnourished status in the category trait), we pooled the RRs for per 1 increment in the CONUT score, as well as malnourished vs. normal nutritional status, respectively. The RRs (logarithmically transformed) and their corresponding standard errors (SEs) were pooled by the inverse variance approach. In case outcomes were presented as odds ratios (ORs) or hazard ratios (HRs), they were regarded as an approximate RR and used in the meta-analysis (22).

Heterogeneity among studies was evaluated with the *I*^2^ statistic, an *I*^2^ value of <50% or *P* for heterogeneity <0.1 was considered an indication of no-significant heterogeneity observed among the studies. However, even when no-significant heterogeneity was shown, we combined the results using the DerSimonian and Laird random-effects models over the fixed effects model, considering that, to some extent, both clinically and methodologically were unavoidable (for example, cohort design, the definition of HF, and adjustment of potential confounders) (23). In case of no heterogeneity, the results of fixed and random effects models are the same, while, if there was significant heterogeneity among the included studies, the random-effects model would be more conservative. To further test the stability of the results, we conducted sensitivity analyses by changing the statistical models from random-effects models to fixed-effects models. We also performed sensitivity analyses by deleting one study each time and recalculating the pooled results. The Publication bias was accessed by inspecting the funnel plot for the outcomes. All the statistical analyses were performed with RevMan 5.3 (The Cochrane Collaboration, Copenhagen, Denmark). A *P* value <0.05 is considered statistically significant.

## Results

### Baseline characteristics of the included studies

**Figure 1:**
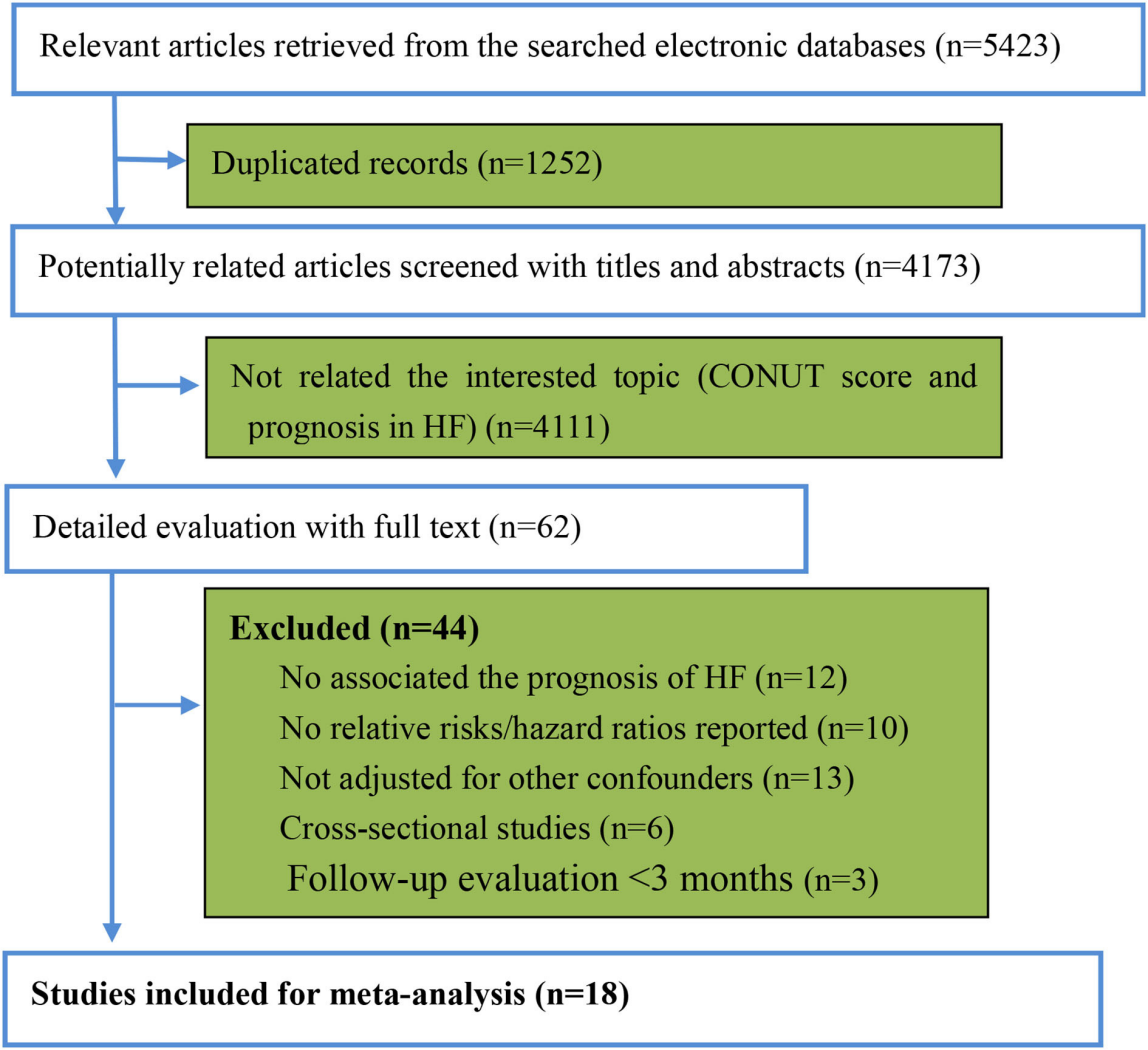
The review flow of the retrieved studies. CONUT, controlling nutritional status; HF: heart failure.

After searching the electronic databases, we retrieved 5,423 potentially related article items. The duplicate items with identical titles, authors, publication journals, and years were deleted. Two investigators (XH and YH) independently screened the titles and abstracts. Then, 62 potentially related full articles were reviewed, and 18 studies were finally included in the pooled analysis according to the pre-defined criteria (Figure 1) (12–16, 24–36). There were 12,532 participants with HF in the included studies, with a median follow-up duration of 32.5 months. The median age of the patients was 70.5 years old, and 35.4% were women. The baseline characteristics of the participants are presented in Table 2. According to the NOS assessment of observational studies, five included studies were graded as fair quality and 13 studies were as good quality (Supplementary File 1). The adjusted confounders in the included studies are summarized in Supplementary File 2.

**Table 2:** Baseline characteristics of the included studies.

**Study**	**Country**	**Cohort design**	**HF type**	**Nutritional status by CONUT score**	**Sample size (** ***n*** **)**	**Female (%)**	**Mean age (years)**	**Follow-up (months)**	**Outcome**
Narumi et al. (25)	Japan	Prospective	CHF	Malnourished (≥5)	388	40.0%	69.6	28.4	MACE
Nochioka et al. (24)	Japan	Prospective	CHF	Continuous variable Malnourished (≥2)	3,421	28.4%	66.9	34.7	All-cause mortality MACE
Nakagomi et al. (26)	Japan	Prospective	CHF	Malnourished (≥3)	114	25.4%	66.0	67.5	MACE
Iwakami et al. (16)	Japan	Prospective	AHF	Continuous variable Malnourished (≥2)	635	38.0%	75.0	27.0	All-cause mortality
La Rovere et al. (28)	Italy	Prospective	AHF	Continuous variable Malnourished (≥2)	466	14.0%	61.0	12.0	All-cause mortality
Nishi et al. (27)	Japan	Retrospective	AHA	Continuous variable Malnourished (≥2)	482	38.2%	71.7	45.1	All-cause mortality
Sze et al. (15)	UK	Prospective	AHF	Continuous variable	265	38.0%	82.0	19.9	All-cause mortality
Shirakabe et al. (14)	Japan	Retrospective	AHF	Malnourished (≥2)	458	34.0%	76.0	12.0	All-cause mortality
Yoshihisa et al. (29)	Japan	Retrospective	AHF	Continuous variable	1,307	39.4%	66.5	38.2	All-cause mortality
Alvarez-Alvarez et al. (30)	Spain	Retrospective	CHF with CRT	Malnourished (≥2)	302	22.5%	70.0	50.4	MACE
Hamada et al. (31)	Japan	Retrospective	CHF	Malnourished (≥5)	67	41.8%	85.0	12	MACE
Chien et al. (13)	China	Retrospective	CHF/HFpEF	Continuous variable Malnourished (>3)	1,120	60.6%	77.2	41.8	All-cause mortality MACE
Uemura et al. (32)	Japan	Retrospective	AHF	Continuous variable Malnourished (≥2)	170	40.6%	67.6	36.5	MACE
Komorita et al. (33)	Japan	Prospective	CHF/HFpEF	Continuous variable	506	45.3%	71.6	50.0	MACE
Sze et al. (10)	UK	Prospective	CHF	Continuous Malnourished (≥2)	467	33.0%	76.0	18.5	All-cause mortality MACE
Ikeya et al. (34)	Japan	Retrospective	CHF with CRT	Continuous Malnourished (≥5)	263	23.2%	69.0	31.0	All-cause mortality
Lu et al. (35)	China	Prospective	AHF	Malnourished (≥2)	396	28.5%	59.8	34.0	All-cause mortality
Takada et al. (36)	Japan	Prospective	AHF	Malnourished (≥2)	1,705	36.0%	71.0	17.5	MACE

AHF, acute heart failure; CONUT, controlling nutritional status; CHF, chronic heart failure; CRT, cardiac resynchronization therapy; HF, heart failure; HFpEF, heart failure with preserved ejection fraction; MACE, major adverse cardiac events.

### Association between CONUT score and risk of all-cause death in HF

**Figure 2:**
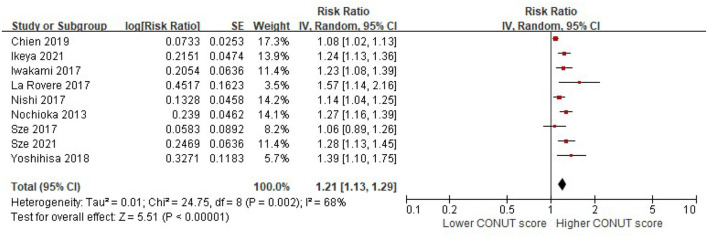
A forest plot of comparison: All-cause mortality in patients with HF associated with per-1 increase of the CONUT score. CONUT, controlling nutritional status; HF, heart failure.

When the CONUT score was reported as a continuous index, we observed that a higher CONUT score was associated with a higher risk of all-cause mortality in patients with HF after adjusting for multiple prognostic factors (per 1 increment of the CONUT score: RR, 1.21, 95% CI, 1.13–1.29, Figure 2). However, significant heterogeneity was observed in the included studies (*I*^2^ = 68%, *P* = 0.002).

**Figure 3:**
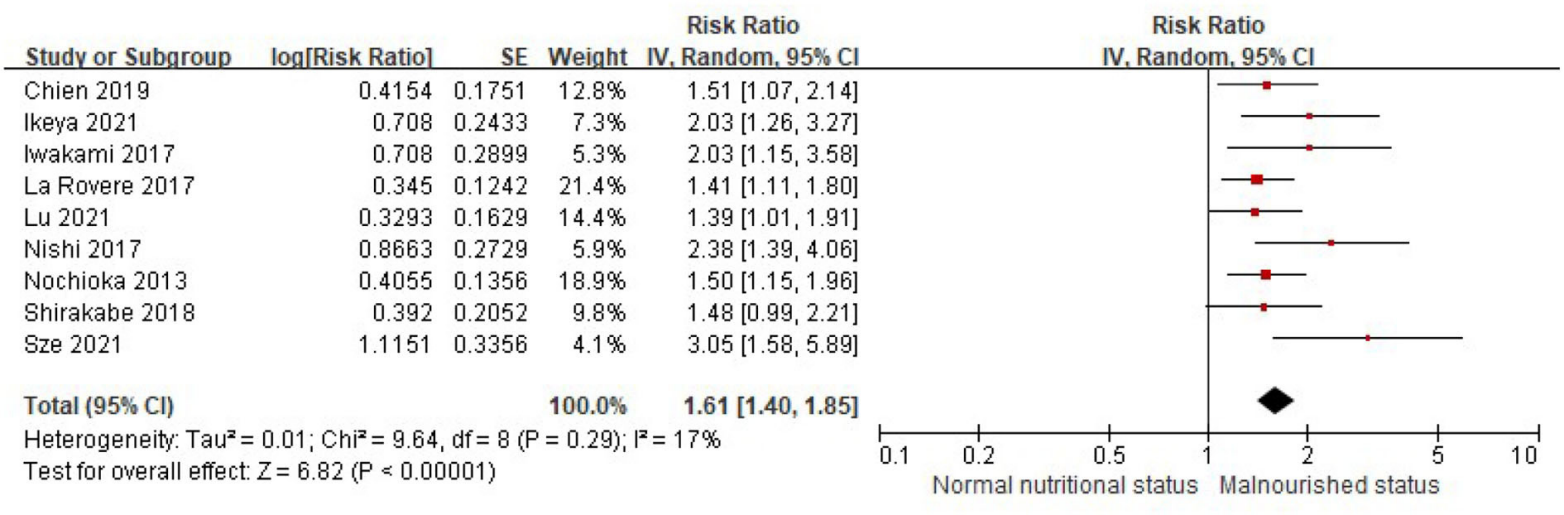
A forest plot of comparison: All-cause mortality in patients with HF associated with malnutrition status defined by the CONUT score. CONUT, controlling nutritional status; HF, heart failure.

**Figure 4:**
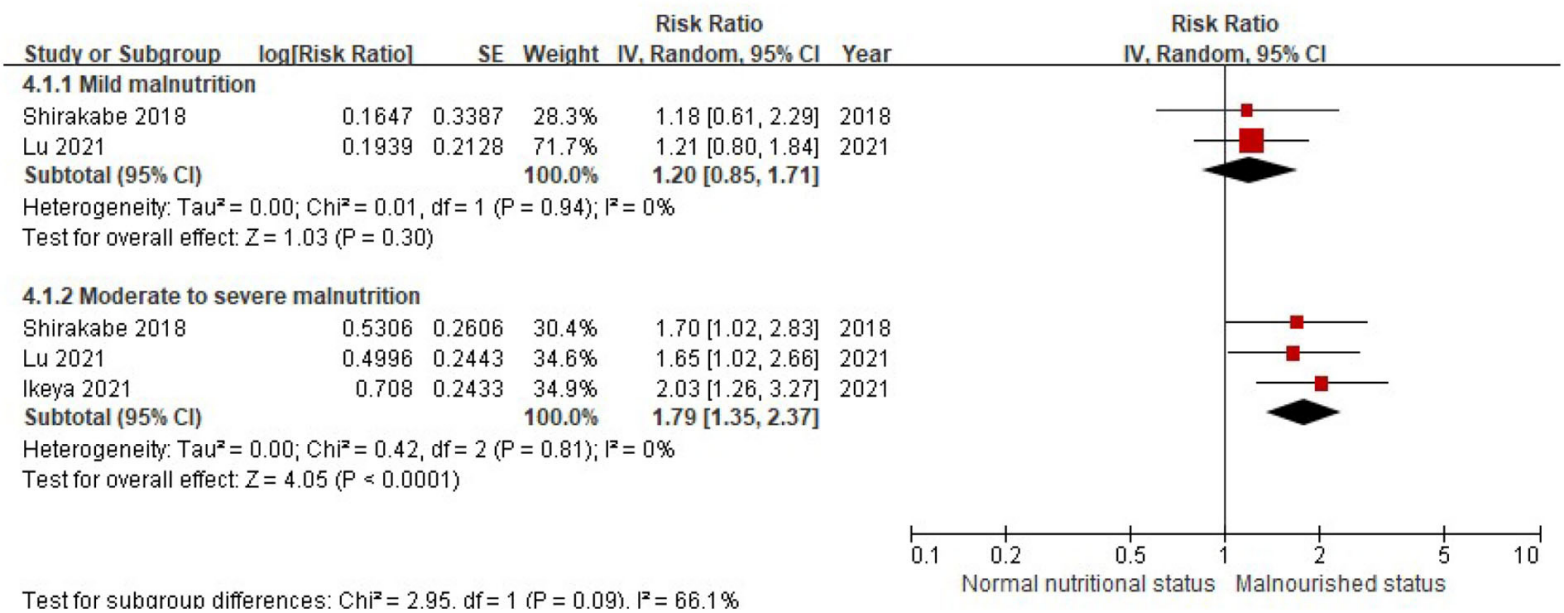
A forest plot of comparison: All-cause mortality in patients with HF associated with different levels of malnourished status defined by the CONUT score. CONUT, controlling nutritional status; HF, heart failure.

When the CONUT score was divided into the normal nutritional status and malnourished status, the patients with a higher CONUT score (malnourished) were associated with a 61% increased risk of all-cause death in HF (RR, 1.61, 95% CI, 1.40–1.85), compared with those with normal nutritional status (a lower CONUT score) (Figure 3) after being adjusted for other prognostic factors. No significant heterogeneity was observed in the included studies (*I*^2^ = 17%, *P* = 0.29). Furthermore, the increased risk of all-cause mortality was only observed in those with moderate to severe malnutrition (the CONUT score ≥5; RR, 1.79; 95% CI, 1.35–2.37), but not in those with mild malnutrition (the CONUT score, 2–4; RR, 1.20; 95% CI,.85–1.71) (Figure 4).

### Association between CONUT score and risk of MACEs in HF

**Figure 5:**
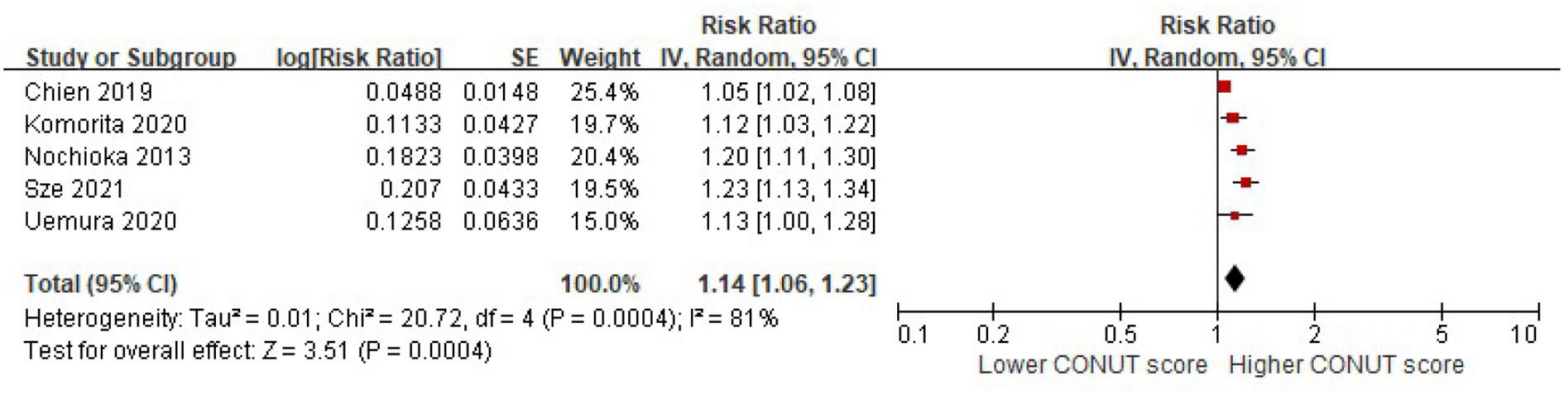
A forest plot of comparison: The risk of MACEs in patients with HF associated with per-1 increase of the CONUT score. CONUT, controlling nutritional status; HF, heart failure; MACEs, major adverse cardiac events.

The patients with higher CONUT scores were associated with a higher risk of MACEs in the patients with HF after being adjusted for multiple prognostic factors (per 1 increment of the CONUT score: RR, 1.14; 95% CI, 1.06–1.23). Significant heterogeneity was observed in the included studies (*I*^2^ = 81%, *P* < 0.0001) (Figure 5).

**Figure 6:**
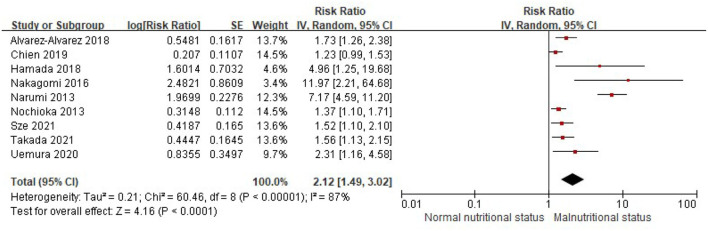
A forest plot of comparison: The risk of MACEs in patients with HF associated with malnutrition status defined by the CONUT score. CONUT, controlling nutritional status; HF, heart failure; MACEs, major adverse cardiac events.

**Figure 7:**
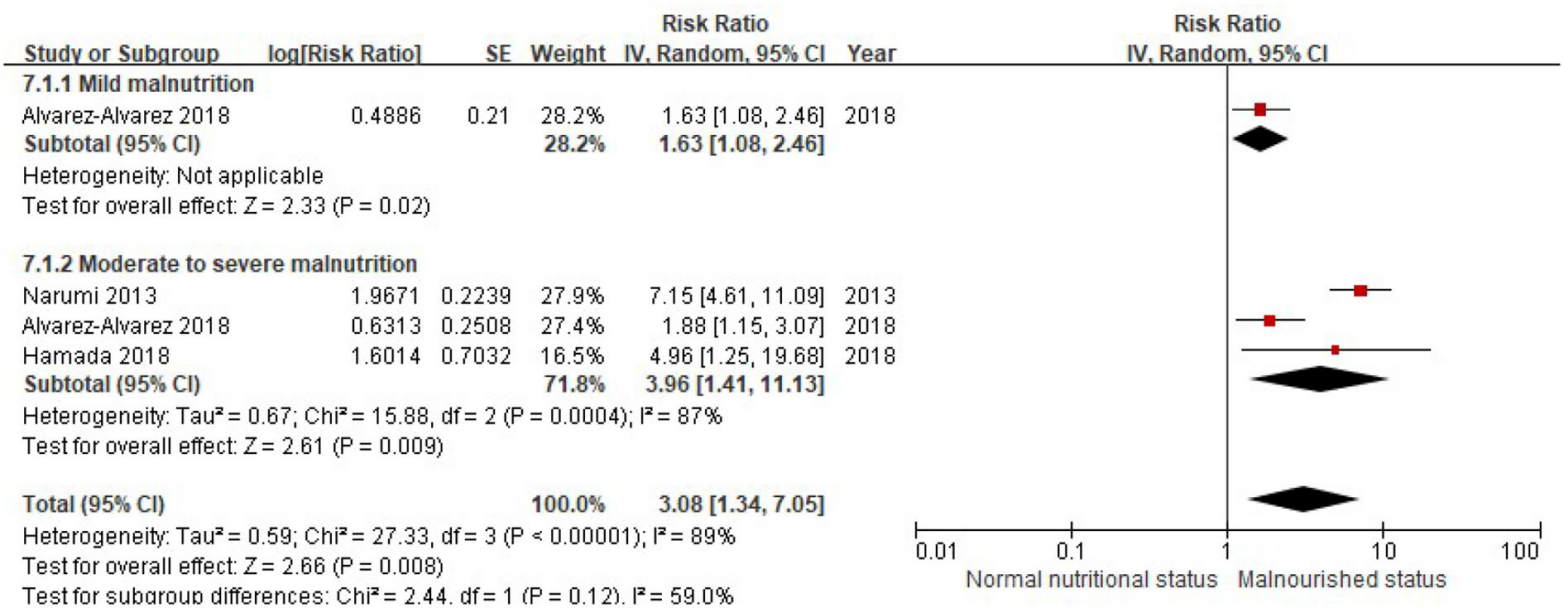
A forest plot of comparison: MACEs in patients with HF associated with different levels of malnourished status defined by the CONUT score. CONUT, controlling nutritional status; HF, heart failure; MACEs, major adverse cardiac events.

Similarly, when the CONUT score was divided into the normal nutritional status and malnourished status, malnourished patients with HF were associated with a 112% increased risk of MACEs (RR, 2.12; 95% CI, 1.49–3.02), compared with those with normal nutritional status in the multivariable-adjusted model (Figure 6), while significant heterogeneity was observed in the included studies (*I*^2^ = 87%, *P* < 0.0001). Compared with patients with HF, with normal nutritional status, those with mild (RR, 1.63; 95% CI, 1.08–2.46) or moderate to severe malnutrition (RR, 3.96, 95% CI, 1.41–11.13) were associated with a high risk of MACEs (Figure 7).

### Sensitivity analyses and publication bias evaluation

The sensitivity analyses confirmed that the association between the CONUT score and the prognosis in the patients with HF did not change with the use of statistical models (fixed-effects models vs. the random-effects models) or recalculation of the RRs by omitting one study at a time. NO significant publication bias was observed for the analyses of all-cause mortality or MACE associated with the CONUT score as a continuous or as a category index by inspection of the funnel plot (Supplementary Files 3–6).

## Discussion

In this meta-analysis, we showed that the CONUT score, which is derived from three commonly detected laboratory biomarkers (e.g., serum albumin, the total cholesterol level, and total peripheral lymphocyte CONUT), is associated with the clinical outcomes in patients with HF. Furthermore, such association was detected when the CONUT score was defined either as a continuous index, or a category divided into the normal nutritional status and malnourished status. These findings support the use of the CONUT score as a screening tool for nutritional status in HF, and guiding the risk stratification, as well as nutritional interventions to improve prognosis in HF.

Similar to our study, a previous meta-analysis by Li et al. (37) included 10 studies involving 5,196 patients with HF, and the results showed that the malnourished patients with HF had an increased risk of follow-up mortality (RR, 2.01; 95% CI, 1.58–2.57). However, the risk of MACEs, including risk of the re-hospitalization, was not evaluated in Li's study. In our meta-analysis, we included a much larger sample size (18 studies with 12,532 participants), which allowed us to perform a much more comprehensive analysis, and our results showed that the risk of MACE in HF was also increased with a higher CONUT score. Furthermore, we found that the worse prognosis (including all-cause mortality and MACEs) was more significant in patients with HF, with moderate to severe malnutrition. Therefore, patients with moderate to severe malnutrition should be emphasized to require more intensive nutritional interventions (e.g., increased protein and energy intake) added to the GDMT, and regular follow-up is needed to improve their prognosis (38).

Several underlying mechanisms may be related to the worse prognosis in HF patients with malnutrition. First, gastrointestinal congestion and gut edema can cause appetite loss and malabsorption (39, 40). Second, the chronic inflammatory state in HF would cause metabolic disturbances, activation of the sympathetic nerve system, and anabolic-catabolic imbalance (41, 42). Third, disturbance of cytokine, adipokines, and metabolites may also play a role in the association between malnutrition and clinical outcomes in HF (43, 44).

Except for the CONUT score, some other simple nutritional indexes had also been proposed in patients with HF (8). For example, the prognostic nutritional index (PNI), which was calculated from the serum albumin and total peripheral lymphocyte count, was reported to be associated with a poor prognosis in patients with acute and chronic HF (46, 47). However, the cut-point for malnutrition by the PNI was inconsistent in different studies, which would hamper its wildly clinical use (48). It had been cautious that the total cholesterol level was included as a component in calculating the CONUT score, which would overestimate the prevalence of malnutrition in patients with HF, as most of them may receive statins treatment and resulted in a lower total cholesterol level (5). In the same cohort of patients, it had been shown that the prevalence of malnutrition would be up to 54% when defined by the CONUT, while only 8% when defined by the PNI (45). However, in patients without statins or other lipid-lowing drug treatment, the inclusion of total cholesterol level may be more comprehensive for evaluating the nutritional status, as it also considered the lipid metabolism (9).

Some limitations in our study should be addressed. First, as discussed above, the CONUT score can be significantly affected by the treatment of statins or other lipid-lowering drugs. However, the proportion of statins treatment was unavailable in most of the included studies. Second, most of the included studies only evaluated the nutritional status at enrollment, but not evaluated the change of nutritional status during the follow-up. However, our results support the conclusion that the baseline nutritional status at enrollment is associated with the prognosis in patients with HF. Third, limited studies were available for the analysis of the different levels of malnutrition and the prognosis. Further studies are needed to document whether mild malnutrition was associated with poor clinical outcomes in HF. Fourth, due to the unavailability of individual patients' data, we cannot perform the analysis of risk discrimination (e.g., c-statistic) and reclassification (e.g., net reclassification improvement or an integrated discrimination index).

## Conclusion

The CONUT score is an easily available nutritional index associated with the clinical outcomes in patients with HF. Further studies are needed to explore whether the CONUT score can be used as a screening tool for nutritional status in HF and guide the nutritional interventions to improve prognosis in HF.

## Data availability statement

The original contributions presented in the study are included in the article/supplementary material, further inquiries can be directed to the corresponding author/s.

## Author contributions

X-WH and BB: research idea and study design. X-WH and J-JL: data acquisition, data analysis/interpretation, and statistical analysis. BB: supervision and mentorship. All the authors contributed important intellectual content during manuscript drafting or revision and accept accountability for the overall work by ensuring that questions pertaining to the accuracy or integrity of any portion of the work are appropriately investigated and resolved.

## Conflict of interest

The authors declare that the research was conducted in the absence of any commercial or financial relationships that could be construed as a potential conflict of interest.

## Publisher's note

All claims expressed in this article are solely those of the authors and do not necessarily represent those of their affiliated organizations, or those of the publisher, the editors and the reviewers. Any product that may be evaluated in this article, or claim that may be made by its manufacturer, is not guaranteed or endorsed by the publisher.
