# Supplementary material for: The controlling nutritional status score and clinical outcomes in patients with heart failure: Pool analysis of observational studies
Source: Front Cardiovasc Med. 2022 Jul 25;9:961141. doi: 10.3389/fcvm.2022.961141 (PMC9357929; doi:10.3389/fcvm.2022.961141)
Supplement: Supplementary file 1 [file Data_Sheet_1.pdf]

Supplementary file 1. Quality Assessment of the Included Studies

Study	Selection (stars awarded)	Comparability (stars awarded)	Outcome (stars awarded)	Quality (total stars)*
Narumi 2013	2	1	2	Fair (5)
Nochioka 2013	3	2	3	Good (8)
Nakagomi 2016	3	2	2	Good (8)
Iwakami 2017	2	1	2	Good (7)
La Rovere 2017	4	1	2	Good (8)
Nishi 2017	3	2	3	Good (8)
Sze 2017	3	2	3	Good (8)
Shirakabe 2018	2	1	2	Good (7)
Yoshihisa 2018	3	1	2	Good (8)
Alvarez-Alvarez 2018	3	1	2	Fair (6)
Hamada 2018	3	2	3	Good (8)
Chien 2019	2	1	2	Good (7)
Uemura 2020	2	1	2	Fair (6)
Komorita 2020	2	1	2	Good (7)
Sze 2021	3	2	3	Fair (6)
Ikeya 2021	3	2	3	Good (8)
Lu 2021	3	2	3	Fair (6)
Takada 2021	3	2	3	Good (8)

* Included studies were graded in quality as good if awarded with ≥7 stars or fair if 4-6 stars.

Supplementary file 2. Adjusted confounders in the included studies

Studies	Adjusted confounders
Narumi 2013	Age, gender, New York Heart Association functional class, and plasma brain natriuretic peptide levels
Nochioka 2013	Age, sex, heart rate, diabetes mellitus, dyslipidemia, history of cancer, current or former smoking, LVEF, BNP levels, anemia, CKD, and treatment (ACEI, ARB, and β-blocker).
Nakagomi 2016	Age, BMI, mCIMT, LVEF, eGFR, CRP, BNP, Hb, TNF-a, and β-blocker, spironolactone.
Iwakami 2017	All potential confounders, some of which were represented by the OPTIMIZE-HF nomogram to avoid overfitting; OPTIMIZE-HF nomogram, hemoglobin, history of malignancy, and statin use.
La Rovere 2017	MAGGIC score, 6MWT
Nishi 2017	Age, sex, CONUT scores as a continuous variable, previous history of HF hospitalization, log BNP, and use of therapeutic agents at admission (tolvaptan and aldosterone antagonists)
Sze 2017	Age, sex, haemoglobin, atrial fibrillation, log NTproBNP, creatinine, sodium, recurrent falls, and the presence of ischaemic heart disease
Shirakabe 2018	Age, SBP, HR, creatinine, total bilirubin, sodium, CRP, hemoglobin, LVEF
Yoshihisa 2018	Age, sex, systolic blood pressure, heart rate, New York Heart Association class, presence of ischaemic aetiology, hypertension, diabetes,

	dyslipidemia, chronic kidney disease, anaemia, atrial fibrillation, B-type natriuretic peptide and LVEF
Alvarez-Alvarez 2018	Age, sex, ischaemic aetiology, atrial fibrillation, sodium, eGFR, Hb, Lad, ACEI/ARB
Hamada 2018	Age ⩾85 years (median), New York Heart Association functional class III/IV at discharge, hypertension, stroke, complete right bundle branch block, beta-blocker
Chien 2019	Age,BMI, sex, prior heart failure, hypertension, cardiovascular disease, diabetes, SBP, heart rate, and atrial fibrillation, hyperlipidaemia, estimated glomerular filtration rate, and brain natriuretic peptide.
Uemura 2020	Age, gender, BMI, history of admission due to heart failure, history of coronary artery disease, hemoglobin, eGFR, and LVEF
Komorita 2020	Age, previous hospitalization for HF, diabetes mellitus, ln-BNP
Sze 2021	Age, BMI, AF compared with sinus rhythm, NYHA, Charlson score, log[NT-proBNP], Hb, and eGFR
Ikeya 2021	Age, sex and liver function parameters (total bilirubin, aspartate aminotransferase, alanine aminotransferase, and γ-glutamyl transferase).
Lu 2021	Age, sex, smoking, SBP, DBP, UA, spartate aminotransferase, NT-proBNP, NYHA, LVEF, LVESD, LVEDD, AF
Takada 2021	Age, BMI, NYHA, atrial fibrillation, DM, history of CABG, LVEF, SBP, heart rate, BNP, eGFR, anaemia, serum sodium, daily furosemide dose

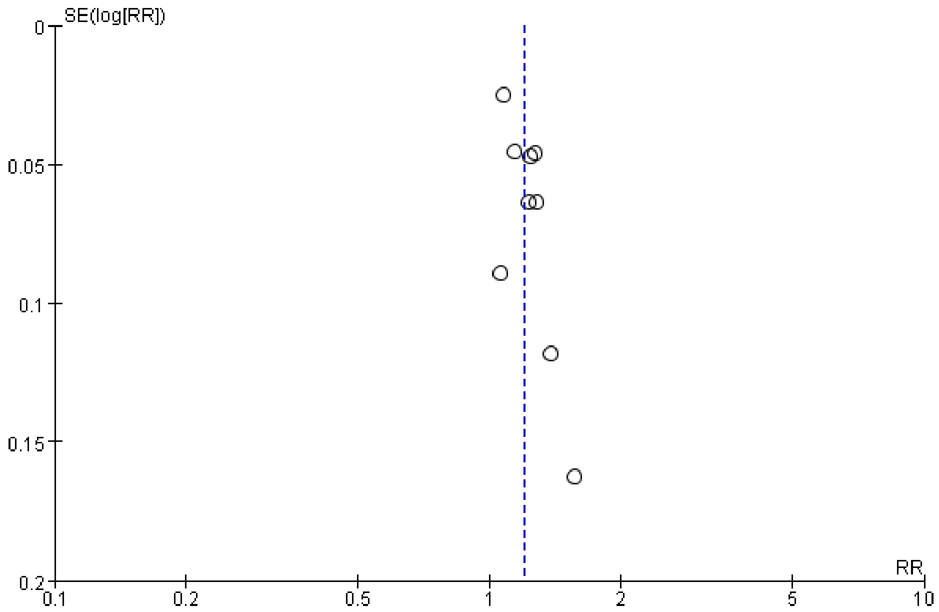

Supplementary file 3. Publication bias evaluated by funnel plot: all-cause mortality in HF patients associated with Per 1 increase of COUNT score

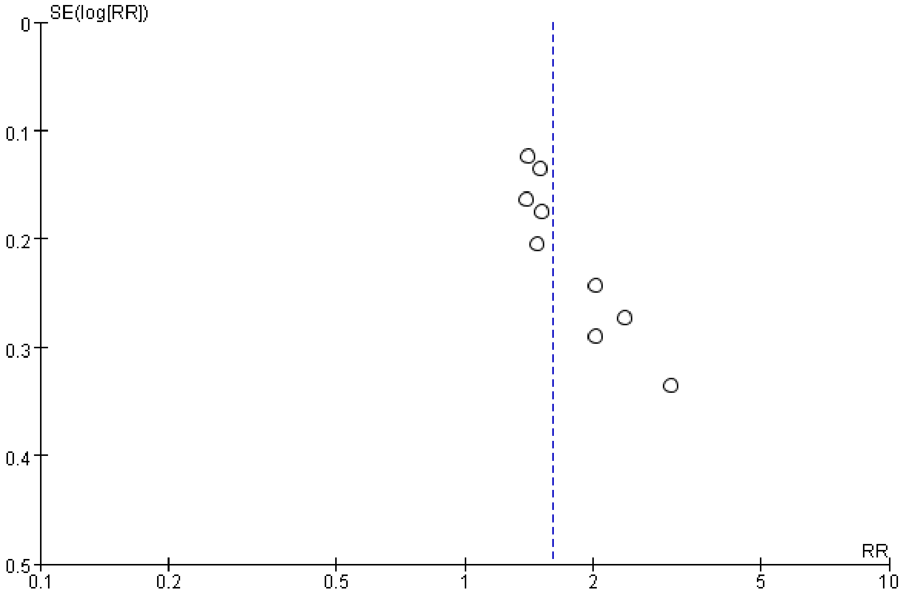

Supplementary file 4. Publication bias evaluated by funnel plot: All-cause mortality in HF patients associated with malnutrition status defined by COUNT score

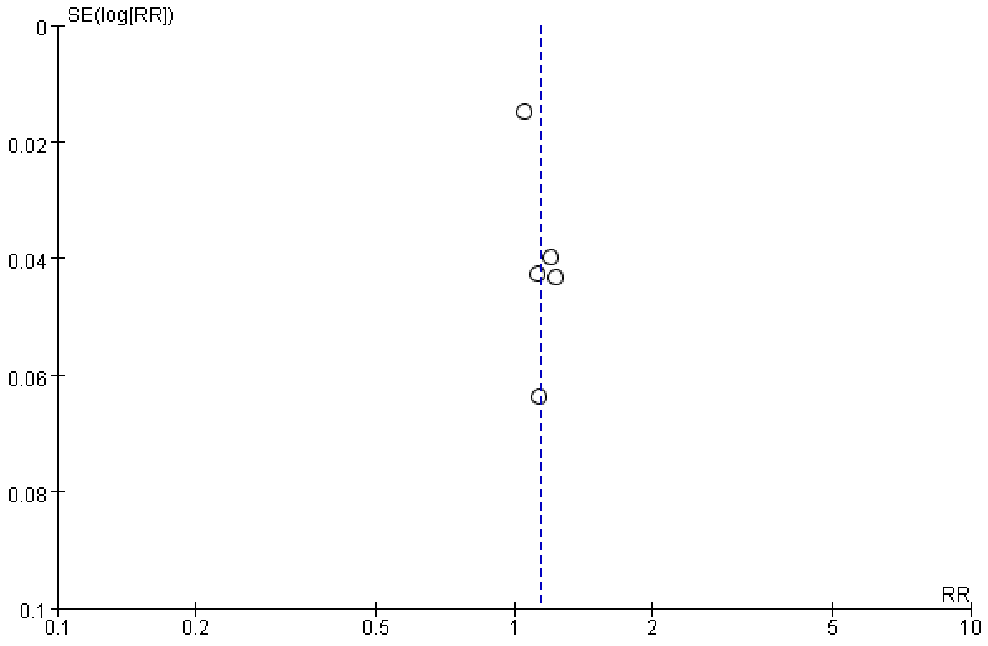

Supplementary file 5. **Publication bias evaluated by funnel plot: MACEs in HF patients associated with Per 1 increase of COUNT score**

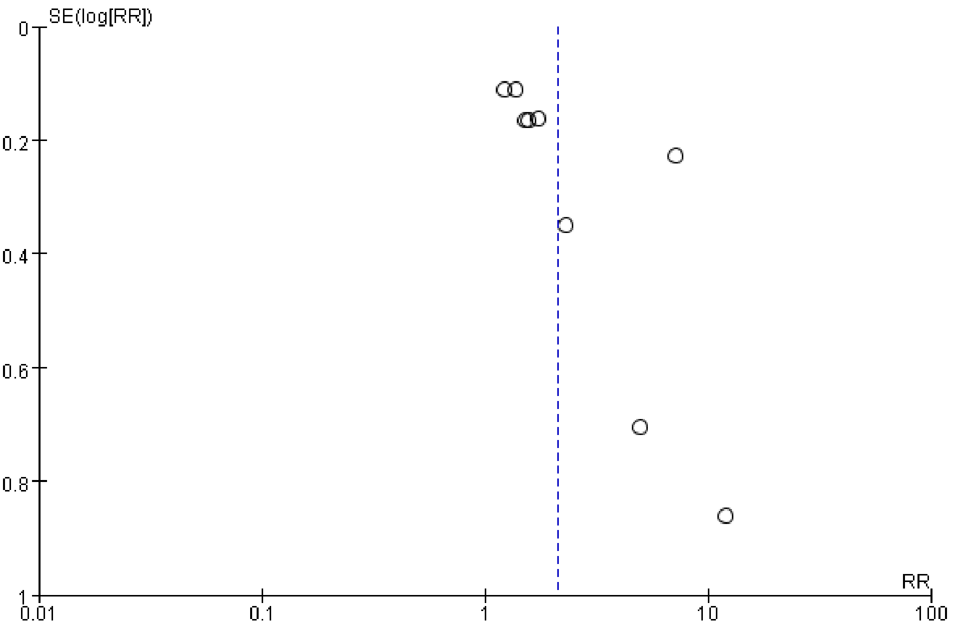

Supplementary file 6. **Publication bias evaluated by funnel plot: MACEs in HF patients associated with malnutrition status defined by COUNT score**
